# Pathogenic Pathways and Therapeutic Approaches Targeting Inflammation in Diabetic Nephropathy

**DOI:** 10.3390/ijms21113798

**Published:** 2020-05-27

**Authors:** Sandra Rayego-Mateos, José Luis Morgado-Pascual, Lucas Opazo-Ríos, Melania Guerrero-Hue, Cristina García-Caballero, Cristina Vázquez-Carballo, Sebastián Mas, Ana Belén Sanz, Carmen Herencia, Sergio Mezzano, Carmen Gómez-Guerrero, Juan Antonio Moreno, Jesús Egido

**Affiliations:** 1 Maimonides Biomedical Research Institute of Cordoba (IMIBIC), University of Cordoba, 14004 Cordoba, Spain; mel10anie@gmail.com (M.G.-H.); crisgcomplutense@gmail.com (C.G.-C.); 2 Renal, Vascular and Diabetes Research Laboratory, IIS-Fundación Jiménez Díaz, Universidad Autónoma de Madrid, 28040 Madrid, Spain; biomorgui@hotmail.com (J.L.M.-P.); lucasopazo78@gmail.com (L.O.-R.); cvazqu01@ucm.es (C.V.-C.); smas@fjd.es (S.M.); asanz@fjd.es (A.B.S.); carmen.herencia@quironsalud.es (C.H.); cgomez@fjd.es (C.G.-G.); jegido@fjd.es (J.E.); 3 Spanish Biomedical Research Centre in Diabetes and Associated Metabolic Disorders (CIBERDEM), 28040 Madrid, Spain; 4 Laboratorio de Nefrología, Facultad de Medicina, Universidad Austral de Chile, Valdivia 5090000, Chile; mezzano.sergioa@gmail.com; 5 Department of Cell Biology, Physiology and Immunology, University of Cordoba, 140471 Cordoba, Spain; 6 Hospital Universitario Reina Sofía, 14004 Córdoba, Spain

**Keywords:** inflammation, type 2 diabetes, diabetic nephropathy, chronic kidney disease, inflammation, drugs, and therapy

## Abstract

Diabetic nephropathy (DN) is associated with an increased morbidity and mortality, resulting in elevated cost for public health systems. DN is the main cause of chronic kidney disease (CKD) and its incidence increases the number of patients that develop the end-stage renal disease (ESRD). There are growing epidemiological and preclinical evidence about the close relationship between inflammatory response and the occurrence and progression of DN. Several anti-inflammatory strategies targeting specific inflammatory mediators (cell adhesion molecules, chemokines and cytokines) and intracellular signaling pathways have shown beneficial effects in experimental models of DN, decreasing proteinuria and renal lesions. A number of inflammatory molecules have been shown useful to identify diabetic patients at high risk of developing renal complications. In this review, we focus on the key role of inflammation in the genesis and progression of DN, with a special interest in effector molecules and activated intracellular pathways leading to renal damage, as well as a comprehensive update of new therapeutic strategies targeting inflammation to prevent and/or retard renal injury.

## 1. Introduction

Diabetic nephropathy (DN) is a common complication of type 1 and type 2 diabetes (T1DM and T2DM). DN is the leading cause of chronic kidney disease (CKD), increasing the morbidity and mortality of diabetic patients [1]. DN morphological abnormalities include early glomerular hypertrophy, glomerular basement membrane (GBM) thickening, podocyte depletion, mesangial matrix expansion, and tubular damage. In later phases, renal alterations include glomerulosclerosis and tubulointerstitial fibrosis, clinically characterized by the loss of renal function with or without albuminuria and progression to end-stage renal disease (ESRD) [2,3].

DN is a multifactorial disease characterized by the complex interaction of hemodynamic and metabolic factors, including high glucose blood levels, advanced glycation end-products (AGEs), and activation of the renin–angiotensin–aldosterone system (RAAS) [4]. Therefore, the current management of diabetic patients focuses on tight glycemic control and antihypertensive/lipid-lowering therapies; however, these interventions do not prevent the progression of CKD in a large proportion of patients [5,6]. For that reason, the search for novel therapeutic approaches against DN is an area of paramount importance. 

**Figure 1:**
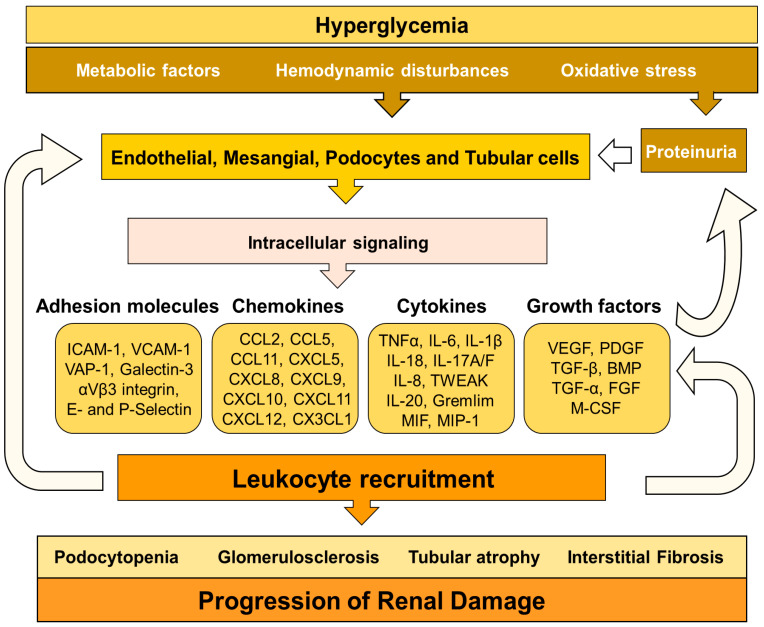
Mechanisms involved in inflammation, tissue injury and progression of renal damage in diabetic nephropathy (DN).

Inflammation is a mechanism activated in response to harmful conditions to maintain tissue homeostasis and integrity. However, chronic activation of the inflammatory response triggers collateral injurious effects [7]. Although hyperglycemia, oxidative stress, and RAAS activation are the driving forces for renal damage associated with diabetes, numerous pieces of evidence point out the key role of inflammation in the development and progression of diabetic complications [8] (Figure 1). Thus, in the last years, the modulation of the inflammatory response has emerged as a potential strategy to decrease diabetic kidney disease [9,10]. These therapeutic approaches are based on inhibition of, adhesion molecules, chemokines, cytokines, immune cells, and intracellular signaling pathways involved in the inflammatory response. This manuscript aims to review the role of inflammation in DN-mediated kidney injury, with a special focus on immune cells, immunoinflammatory mediators, and cell signaling pathways, as well to fully address novel anti-inflammatory therapies to prevent and/or retard the progression of renal damage in this pathological context.

## 2. Mechanisms Triggering DN-Associated Inflammation

In DN, hyperglycemia leads to the expression of inflammatory mediators (chemokines and cytokines) by injured glomerular and tubular cells, contributing to renal damage by different mechanisms: mesangial proliferation, podocyte/tubular damage and leukocyte infiltration [11,12]. These proinflammatory molecules also induce extracellular matrix deposition and differentiation/proliferation of myofibroblast through different signaling pathways, such as NF-κB, JAK/STAT, TGFβ/Smad, among others [13,14,15].

In DN, both hyperglycemia and hemodynamic abnormalities elicit glomerular hyperfiltration, mechanical stress, glycocalyx dysfunction, and endothelium activation [4,16]. Consequently, endothelial cells upregulate the expression of adhesion molecules, glycosaminoglycans and chemokines, which are involved in the infiltration of leukocytes toward the renal interstitium [17]. Chemokines promote the activation of integrins by leukocytes, allowing the interaction of these cells with the endothelial adhesion molecules [18]. This interaction results in the adhesion of inflammatory cells to the endothelium and further transmigration to other tissues [19]. Once infiltrated in the inflammatory foci, leukocytes promote renal damage by two different mechanisms: (1) direct interaction and activation of glomerular and tubular cells, and (2) releasing chemokines, cytokines, and profibrotic factors that activate stromal renal cells. Then, these stromal renal cells secrete additional chemokines promoting further infiltration of leukocytes. All these events amplify the inflammatory response in a positive feedback loop that enhances renal damage [9,12]. Inflammatory molecules induce vascular remodeling, endothelial dysfunction, extracellular matrix deposition, mesangial proliferation, podocyte and tubular death, GBM thickness and glomerulosclerosis, which are hallmarks of diabetic kidney disease [2,20].

There is a close and retroactive relationship between oxidative stress and inflammation in DN [21,22,23,24]. Thus, experimental models of DN have demonstrated that the production of cytokines is partially promoted by hyperglycemia-mediated oxidative stress [25,26]. Studies in patients have demonstrated that glucose administration increased IL-6 or IL-18 plasma concentration, an effect that was reduced with the antioxidant glutathione [27]. In this line, a strong and independent relationship between oxidative stress markers and cell adhesion molecules (ICAM-1) and other inflammatory mediators (CCL2) was found in T1DM and T2DM patients [28,29]; however, vitamin E administration abolished this association [30,31,32]. The induction of proinflammatory factors by oxidative stress can occur through the activation of NF-κB and the activator protein-1 (AP-1), two key transcription factors mediating inflammatory response during DN, as we will fully describe in the next sections of this review [25,33,34,35]. 

## 3. Immune Cells and DN

Cells of the innate and adaptive immune response are involved in DN. In the next section, we will fully address the role of these immune cells in this clinical condition.

### 3.1. Macrophages

Macrophages are early recruited during the genesis of renal injury in diabetes and are also associated with the progression of the disease [36,37]. Macrophage infiltration is mediated by several chemokines, including chemokine-CC motif ligand 2 (CCL2), CCL5, CXCL1, CXCL16, and CXCL10 chemokines receptors and adhesion molecules (ICAM-1 and E-selectin) [38,39,40]. 

Macrophages produce different molecules triggering renal injury in DN, including reactive oxygen species, proinflammatory cytokines (IL-1β and TNF-α), chemokines (CCL2), factors of the complement system, and metalloproteinases [38,39,41]. Macrophage activation and accumulation positively correlate with hyperglycemia, glomerular/tubular damage, endothelial activation, and decline of renal function [36]. Thus, macrophages induce proteinuria and glomerulosclerosis [42]. In a model of progressive DN, renal macrophage infiltration correlated with renal chemokine expression, such as CCL2, migration inhibitory factor, osteopontin, and monocyte colony-stimulating factor [36]. Moreover, preclinical studies in models of DN showed that glomerular and interstitial macrophages positively expressed activation markers such as sialoadhesin and inducible nitric oxide synthase [38]. Macrophages also release profibrotic mediators (PDGF and TGF-β) that elicit mesangial-mediated fibronectin production and fibroblast/mesangial proliferation [43]. 

Lipid abnormalities are frequent in diabetic patients. Hyperlipidemia induces macrophage-derived foam cells formation. Thus, macrophages exposed to serum samples from T2DM patients were transformed into foam cells [44]. An increased presence of foam cells was observed in the renal glomerulus of diabetic mice fed on a hypercholesterolemic diet [45]. Lipid lead macrophages release a high number of cytokines, thus contributing to the inflammatory process. 

In the early and middle stages of DN, infiltrated macrophages are derived from recruited monocytes, which undergo polarization/differentiation towards a proinflammatory M1 phenotype rather than anti-inflammatory M2 phenotype. Indeed, macrophages isolated from diabetic kidneys showed the greatest expression of M1 markers (Ly6C, IL-6 and CCR2) than M2 markers (CD206 and CD163) [41]. In DN, polarization toward the M1 phenotype is related to the activation of several intracellular signaling pathways (NF-κB, JAK/STAT, SREBP-1) in response to TNF-α, glucose, and AGEs production [46,47]. The M1 macrophages produce TNF-α, accelerating the inflammatory process in DN [48]. Moreover, M1 macrophages directly disturb podocyte integrity [41]. Specific deletion of cyclooxygenase-2 in macrophages regulated the process of macrophage differentiation in DN [49]. The heterogeneity of macrophage phenotype and function ultimately determines the outcome of DN [50]. M2 macrophages secrete IL-10, with potent anti-inflammatory effects, such as the activation of HO-1 and the inhibition of TNF-α production [51]. Therefore, induction to the M2 phenotype has been one of the key goals of immune-based treatments for DN. 

### 3.2. Dendritic Cells

Dendritic cells (DCs) are mononuclear phagocytes that reside in many organs, including the kidney. These antigen-presenting cells show different phenotypes and functions depending on the local microenvironment. In the kidney, DCs act as a bridge between innate and adaptive immunity [52] and participate in the tubulointerstitial immune cell cross-talk during the progression of DN [53,54]. Matured DCs produce cytokines and costimulatory molecules and activate T lymphocytes and renal macrophages, thus mediating inflammation and kidney damage. Experimental immunomodulatory strategies, such as mesenchymal stem cell transplantation and Fms-like tyrosine kinase 3 inhibitor, reduce the population and maturation of DCs and ameliorate inflammation and CKD [55,56]. However, there is still limited information about the distinct functions of DCs’ subsets in the pathogenesis of DN. 

### 3.3. T Lymphocytes

Several studies have demonstrated the increased presence of activated CD4+, CD8+ T cells in renal interstitium of patients with diabetes [57]. These T cells release IFN-γ and TNF-α, as noted in diabetic mice, thus contributing to the promotion of inflammation by activation of macrophages and endothelial cells [57]. Specifically, CD4+T cells also enhance fibrosis by activating fibroblasts [58]. The number of CD4+ T cells in diabetic kidneys correlated positively with proteinuria levels [57]. In the same line, induction of DN in mice without T and B lymphocytes (Rag1-/- mice) showed that lymphocytes activate glomerular macrophages, induce podocyte injury, and increase albuminuria [59]. Pharmacological inhibition of T-cell activation with CTLA4-Fc abatacept also reduced albuminuria in mice with DN [60]. 

CD4+ T cells can differentiate into different cell-subtypes, including T-helper subsets (Th1/Th2/Th17) and regulatory T cells (Tregs). Th1 and Th17 cells have been specifically associated with renal damage in DN. These inflammatory cells release chemokines in response to IL-1β, IL-6, and TGF-β, which are frequently found in diabetic kidneys [48]. Targeting Th17 cells by mycophenolate mofetil attenuated albuminuria and tubulointerstitial fibrosis in mice with DN [61]. On the other hand, depletion of Tregs with anti-CD25 mAb exacerbates diabetic-associated renal injury in mice, whereas the adoptive transfer of Tregs had the opposite effect [62].

### 3.4. B Lymphocytes

B-cells are found in the glomeruli of diabetic patients and experimental models of DN, suggesting the involvement of these cells in this pathological setting [57,59]. The pathogenic role of B-cells in DN is based on their capacity to produce immunoglobulins (predominantly IgG and IgM isotypes) against neoantigens formed as a consequence of diabetes, such as AGE–LDL, malondialdehyde-LDL and oxidized-LDL [63,64]. These immunoglobulins activate specific Fc receptors and the complement system [65,66]. In this line, increased IgG and C3 glomerular deposits were observed in experimental DN [36,59]. Moreover, the presence of IgG in renal biopsies from diabetic patients was associated with worse outcomes [67]. Interestingly, diabetic mice lacking IgG Fc receptors had reduced renal damage, inflammation, and fibrosis [68]. Therefore, strategies aimed to reduce Fc receptor signaling in DN may be of interest.

### 3.5. Mast Cells

Mast cells are myeloid-derived immune cells with an important role in many autoimmune and inflammatory diseases. Mast cells infiltrate the diabetic kidney in all stages of the disease, and their number and degranulation levels increase in line with the progression of the disease in patients [69,70]. The anaphylatoxin C3a triggers mast cells activation and chemotaxis toward inflammatory foci, and probably may be responsible for their recruitment in DN [71]. Mast cells are also able to promote renal fibrosis via SCF/c-kit signaling pathway and induce tubular interstitial injury by increasing the production of proteases and inflammatory mediators, such as chymase, renin, TGF-β1 and TNF-α [70,71]. Inhibition or reduction of chymase-positive mast cells, ameliorated albuminuria in db/db mice [72]. A clinical trial with the chymase inhibitor BAY1142524 in patients with DN is now completed, but no results have been yet posted (NCT03412006).

### 3.6. Neutrophils

There is limited information about the possible role of neutrophils in DN. Nevertheless, neutrophils from T1DM and T2DM patients have a higher capacity to adhere to endothelium as compared to those from normo-albuminuric patients [73]. Additionally, neutrophils release high amounts of ROS and TNF-α, thus promoting endothelial damage and, therefore, accelerate DN-mediated renal damage [74,75]. Also, increased neutrophil to lymphocyte ratio has been associated with albuminuria in diabetic patients [76].

## 4. Inflammatory Mediators in DN

**Figure 2:**
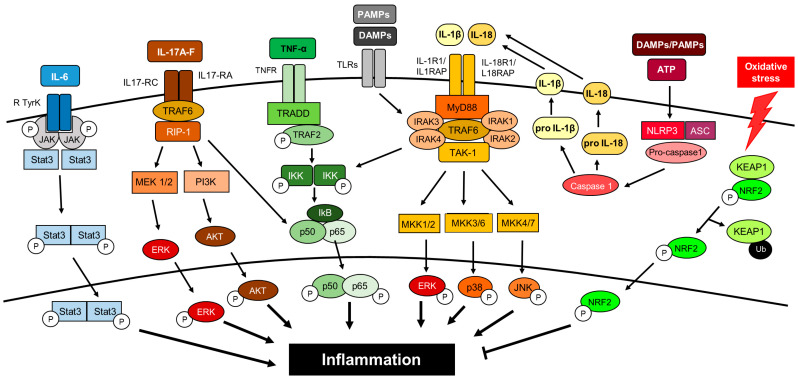
Cytokines and intracellular signaling pathways activating inflammation in DN. This is a simplified view since there are tremendous connections between the different pathways, indicating the complexity of the inflammatory response.

As previously described, cell adhesion molecules, chemokines and cytokines play a key role in renal damage associated with DN. In this section, we will fully address the key role of these molecules in DN-mediated renal damage as well as different therapeutic approaches targeting these proinflammatory molecules in this pathological condition (Figure 2).

### 4.1. Cell Adhesion Molecules

Cell adhesion molecules (CAMs) are implicated in leucocyte trafficking between blood and tissues. These molecules mediate leukocyte rolling on vascular endothelial cells and tightly adhere them to the endothelium to be further recruited to the inflammatory foci [77]. Therefore, CAMs mediate the interaction between leukocytes and endothelial cells, playing a key role in DN. There are many CAMs involved in the onset and development of diabetes-associated renal injury.

Intercellular adhesion molecule 1 (ICAM-1) is a cell adhesion molecule that interacts with macrophage-1 antigen (Mac-1) and lymphocyte function-associated antigen-1 (LFA-1). ICAM-1 expression is upregulated in endothelial cells of glomerular and interstitial capillaries by shear/oxidative stress and cytokines [78,79,80]. Plasma ICAM-1 levels are increased in T1DM and T2DM patients, being associated with urinary albumin excretion rate or microalbuminuria [81,82]. A recent meta-analysis showed that the rs5498 polymorphism is a risk factor for DN in Caucasian T1DM patients [83]. ICAM-1 gene deletion or the use of an anti-ICAM-1 antibody reduced macrophage infiltrate as well as decreased mesangial proliferation, glomerular Col IV expression and albuminuria in different experimental models of diabetes [40,84]. 

Vascular cell adhesion molecule-1 (VCAM-1) was also found elevated in renal vascular endothelium and infiltrating leukocytes in diabetic kidneys. In T2DM patients, increased levels of VCAM-1 correlated with albuminuria [85] and were identified as a prominent mortality risk factor [86].

Vascular adhesion protein-1 (VAP-1) is an endothelial cell-surface oxidase that induces oxidative stress and cellular toxicity [87]. VAP-1 plasma levels are elevated in diabetic patients, being associated to eGFR decline and albuminuria [88,89], and may be related with cardiovascular and cancer mortality risk of these patients [89,90]. A recent phase II clinical trial in diabetic and CKD patients showed that the administration of the oral VAP-1 inhibitor ASP8232 in combination with RAS blockade, delayed progression of renal damage [91]. 

Galectin-3 is a β-galactoside binding lectin that has been related to DN because acts as a receptor for AGEs [92]. Circulating galectin-3 levels increased in line with loss of renal function and was associated with cardiovascular events and mortality in two clinical cohorts (4D and LURIC) [93]. The galectin-3 antagonist GCS-100 was used in diabetic patients with CKD, but results has not been yet posted (NCT02312050).

αVβ3 integrin is expressed by the glomerular endothelium and podocytes [94]. Increased expression of the αVβ3 integrin and its ligand (vitronectin) has been observed in kidneys from diabetic rats [95]. Blockade of the αVβ3 integrin using a monoclonal antibody decreased proteinuria and early histologic changes of diabetic nephropathy in pigs (Table 1) [96]. Moreover, the use of αvβ3 Integrin antagonist (MK-0429) in ZSF1 rats decreases proteinuria, renal fibrosis and collagen accumulation in diabetic damaged kidneys [97]. An ongoing clinical trial evaluates the effect of a αVβ3 integrin antibody in patients with DN, although no results have been published (NCT02251067).

Selectins have been also implicated in DN, and E-selectin is the most studied one in this pathological context [98]. In diabetic mice, E-selectin expression is induced by IL-1β or TNF-α levels being observed in glomeruli, mainly located in endothelial cells [99], and this expression was correlated with the amount of CD14 positive interstitial cells [98]. In T1DM patients, increased E-selectin plasma levels have been reported, correlating with the decline of renal function and cardiovascular risk [100]. In the case of P-selectin, T2DM patients showed increased levels of P-selectin and associated to the severity of disease [101], an effect that was reduced with the combination of ARB olmesartan and the ACEi imidapril [102].

### 4.2. Chemokines

Chemokines are small soluble signaling proteins that can be involved in glomerular and tubulointerstitial inflammation. These molecules are released by resident renal cells recruiting and activating circulating leukocytes [103]. 

Several chemokines (CCL2, CCL5, CCL11, CXCL5, CXCL8, CXCL9, CXCL12, CX3CL1) and chemokine receptors (CCR2, CCR5, CXCR4, CX3CR1) were found to be elevated in kidneys of diabetic mice/rats and in renal biopsies from DN patients [104,105]. In diabetic kidneys, these chemokines and their cognate receptors are expressed by tubular cells and podocytes, as well as infiltrating leukocytes [104,106]. The renal expression of these proteins is progressively increased in line with macrophage infiltration, proteinuria and renal function decline [104]. Consequently chemokine urinary levels, such as CCL2, CXCL5, CXCL8 and CXCL9, are significantly elevated in later phases of diabetic kidney disease [107,108]. A study in patients with DN showed that urinary levels of CXCL9 and CXCL11 mRNA levels correlated with the eGFR decline [109]. CXCL12/SDF-1-α plasma levels were useful for predicting the progression of renal dysfunction in patients with coronary artery disease [110]. Therefore, urinary and/or systemic analysis of chemokine levels may be a useful clinical tool for patient risk stratification. 

The expression of chemokines is directly upregulated by the diabetic harmful environment. Thus, CCL2 is activated by high glucose and AGE via NF-κB in mesangial cells [111]. The role of CX3CL1/CX3CR1 axis in DN has been recently reviewed [112]. CX3CL1 (fractalkine) was found in glomerular and peritubular capillaries, whereas CX3CR1 is expressed by T lymphocytes and activated monocytes/macrophages [113]. Experimental data indicated an important role of this axis in the progression of DN, as well as a direct relation with RAAS [113]. In DN, CX3CL1 pathogenic effects include mesangial cell proliferation via cellular reactive oxygen species (ROS) generation and activation of MAPK signaling pathway [114], extracellular matrix accumulation [115] and podocyte apoptosis [116]. Moreover, the inter-regulation of CX3CL1 and MMP2 mediates the crosstalk between monocytes and mesangial cells in the context of DN [117].

In experimental models of DN, targeting chemokines and its receptors reduced renal inflammation and the subsequent pathological consequences, including oxidative stress, fibrosis, and glomerular damage [36,118]. There are many strategies to modulate the action of chemokines and its receptors, such as the use of specific chemokines receptor antagonists or chemokine inhibitors. For example, the blockade of CXCL12 by the specific inhibitor NOX-A12 diminished podocytopenia and glomerulosclerosis independently of glycemic control or glomerular macrophage infiltration [119]. The pharmacological inhibition of CXCR4 in diabetic rats with the small molecule inhibitor AMD3100 increased albuminuria, reduced VEGF excretion, and accelerated tubular epithelial cell death, indicating that CXCR4 may be a promoter of renal tubular cells survival [106]. In diabetic mice, the injection of a recombinant murine CXCL10 reduced albuminuria, mesangial and peritubular cell expansion, and glomerular hypertrophy [120]. In experimental diabetes, several CCR2 antagonists (such as propagermanium, CCX140-B, RO5234444, RS102895 or RS504393) selectively blocked CCL2-dependent monocyte activation and provided beneficial metabolic effects against DN-mediated podocyte injury and albuminuria [121,122,123,124]. A new area of interest is the inhibition of multiple chemokines at the same time. For example, dual inhibition of CCL2 and CXCL12 with specific RNA enantiomers (CCL2-specific mNOX-E36 and the CXCL12-specific NOX-A12) had additive effects against diabetic-associated renal injury [125]. 

These positive experimental data have been confirmed in human clinical trials. Thus, the CCR2 inhibitor CCX140-B administered for up 52 weeks reduced urinary albumin/creatinine ratio (UACR) in patients with DN [126]. In the same line, inhibition of CCL2/CCR2 signaling with emapticap pegol (NOX-E36) for up 12 weeks showed beneficial effects on urine albumin levels and reduced high blood glucose levels in T2DM patients with residual albuminuria on RAAS blockade [127]. However, no protective effects were observed during 12 weeks of treatment with F-04634817, a dual chemokine CCR2/5 receptor antagonist, in T2DM patients that were on ARBs and/or ACEi therapy [128]. In that study, patients had high levels of albuminuria and low eGFR at baseline, suggesting that the beneficial effects of chemokine targeting may be more effective at earlier stages of DN.

**Table 1:** Selected preclinical studies targeting inflammatory mediators in DN.

Target	Diabetic Model	Strategy	Category	Conclusion	Ref.
**CAM**	SD rats (65 mg/kg)	Anti-ICAM-1 antibody	ICAM-1 antagonist	Prevents glomerular mononuclear cell infiltration.	[84]
	Yorkshire pigs + STZ (50 mg/kg)	Anti-αVβ3 antibody	αVβ3 antagonist	Attenuates proteinuria and renal histological changes.	[96]
	ZSF1 rats	MK-0429	αVβ3 inhibitor	Reduces proteinuria and renal fibrosis	[97]
**Chemokines**	SD rats + STZ (60 mg/kg)	AMD3100	CXCR4 inhibitor	Increases albuminuria and accelerated tubular cell death.	[106]
	db/db mice	NOX-A12	CXCL12 inhibitor	Decreases glomerulosclerosis and albuminuria.	[119]
	db/db mice	Recombinant CXCL10	Mimetic CXCL10	Reduces mesangial matrix expansion, albuminuria, and glomerular hypertrophy.	[120]
	iNOS-Tg mice	Propagermanium	CCR2 antagonist	Decreases mesangial matrix expansion and macrophage infiltration.	[121]
	db/db mice	RS504393	CCR2 antagonist	Ameliorates inflammation, oxidative stress, and fibrosis.	[123]
	db/db mice	CCX140-B	CCR2 antagonist	Reduces albuminuria, glomerular hypertrophy and increases podocyte number.	[124]
**Cytokines**	Wistar rats+ STZ (40 mg/kg)	Infliximab	TNF-α inhibitor	Decreases albuminuria.	[129]
	KK-A(y) mice	Etanercept	TNF-α inhibitor	Improves albuminuria, macrophage infiltrate and CAM expression.	[130]
	SD rats + STZ (65 mg/kg)	Tocilizumab	IL-6 inhibitor	Decreases albuminuria, oxidative stress, inflammation.	[131]
	BKS db/db mice	Anti-IL-1β antibody	IL-1β inhibitor	Improves kidney injury markers and attenuates decline of eGFR.	[132]
	db/db, and Akita mice; STZ (150 mg/kg)	Recombinant IL-17A	Mimetic IL-17A	Prevents fibrosis, podocytes loss, tubular atrophy, and albuminuria.	[133]
	BTBR ob/ob mice	Anti-IL-17A antibody	IL-17A inhibitor	Ameliorates renal function, macrophage infiltration and podocyte loss.	[134]
	Wistar rats + STZ (70 mg kg)	Anti-IL-20 antibody	IL-20 inhibitor	Reduces glomerular area and improves renal functions.	[135]
	db/db mice	ISO-1	MIF inhibitor	Decreases albuminuria, fibrosis and inflammation.	[136]
	Wistar rats + STZ (50 mg kg)	p425	MIF antagonist	Decreases UACR, serum BUN and creatinine.	[137]

**Abbreviations**: CAM: cell-adhesion molecules; BUN: blood urea nitrogen; eGFR: estimated glomerular filtration rate; STZ: streptozotocin; SD: Sprague Dawley; ZSF1: Zucker fatty/spontaneously hypertensive heart failure F1 hybrid.

### 4.3. Cytokines

#### 4.3.1. TNF-α

TNF-α influences the recruitment and activation of leukocytes, exacerbating DN-associated inflammatory response [11,138]. In diabetic kidneys, TNF-α and its receptors TNFR1 and TNFR2, are involved in the synthesis of cytokines, chemokines, growth factors, extracellular matrix proteins and mediate a wide variety of cytotoxic effects on podocytes, mesangial, endothelial and epithelial cells [138,139,140]. Moreover, the serum and urinary levels of TNF-α are increased in patients with DN compared to healthy controls and there is a close relationship with albuminuria in diabetic patients, suggesting TNF-α as a potential prognostic biomarker in DN [140,141,142]. TNF-α receptors can be shed from the extracellular membrane as two soluble proteins, named sTNFR1 and sTNFR2. These molecules were able to predict the loss of eGFR and ESRD in T1DM and T2DM patients [140,143,144]. In a recent study, 194 circulating inflammatory proteins were analyzed in T1DM and T2DM subjects from three independent cohorts, identifying an extremely robust kidney risk inflammatory signature (KRIS), consisting of 17 proteins of the TNFR superfamily members that were associated with a 10-year risk of ESRD [10]. Therefore, these proteins may be new prognostic biomarkers for progressive DN. Some treatments, such as pentoxifylline and ACEi, reduced the TNF-α renal expression in mice and patients with diabetes [139,145]. In those studies, the reduction of TNF-α levels was directly associated with the decline of albuminuria, highlighting a pathogenic role of TNF-α in DN. In the PREDIAN trial, patients with T2DM and CKD (stages 3–4) were treated with pentoxifylline in addition to RAAS inhibitors for 2 years, resulting in a smaller decrease in eGFR and greater reduction of residual albuminuria, coinciding with a marked reduction in urinary TNF-α [146]. Recently, new clinical trials have proposed pentoxifylline as a possible therapeutic agent in this pathological context (NCT03664414; NCT03625648). There are strategies specifically targeting TNF-α in this pathological context. A soluble TNF-α antagonist (TNFR:Fc) reduced urinary TNF-α excretion as well as renal damage associated with DN [147]. Similar results were observed with the monoclonal antibody infliximab, a TNF-α inhibitor that reduced albuminuria in experimental diabetes [129]. Etanercept, a soluble TNFR2 fusion protein targeting TNF-α-TNFR2 pathway, reduced the levels of cell adhesion molecules, macrophage renal infiltrate, and albuminuria in diabetic mice [130]. However, to our knowledge, no clinical studies are targeting TNF-α in the context of renal complications of diabetes.

#### 4.3.2. IL-6

Il-6 is a cytokine that plays a key role in inflammatory pathologies. A recent meta-analysis in patients with DN revealed the essential role of different polymorphisms of IL-6 such as rs1800795, rs1800796, and rs1800797 in the development and progression of DN [148]. The IL-6–174 G allele increased the risk to develop renal complications in T2DM patients [149]. These pieces of evidence suggest that IL-6 may be related to DN. Increased IL-6 urinary levels were found in patients with DN, mainly in those with worse renal outcomes [150]. Some therapies have been developed to inhibit IL-6 signaling pathway [151]. Tocilizumab, a humanized antibody that blocks the IL-6 receptor (IL-6R), attenuated the histopathological changes induced by streptozotocin (STZ) in rats, by decreasing inflammation and oxidative stress [131]. Clinical trials provided metabolic beneficial effects of anti-TNF-α agents (infliximab, etanercept, adalimumab, golimumab, and certolizumab pegol) or IL-6 inhibitor (tocilizumab) on HbA1c, insulin resistance and insulin sensitivity in T2DM patients with psoriasis [152] or rheumatoid arthritis [153].

#### 4.3.3. IL-1β

Interleukin-1β (IL-1β) is one of the most potent molecules of the innate immune system implicated in macro- and micro-vascular complications of diabetes [154,155,156]. This cytokine is highly produced by macrophages and triggers the production of other secondary proinflammatory mediators by renal cells [157]. Indeed, IL-1β induced tubulointerstitial fibrosis throughout the activation of the MYC transcription factor, with further dysregulation of glycolysis and matrix production [158]. Several studies described that IL-1β causes endothelial cell damage in resistance arteries and identified its deleterious effect due to the NADPH oxidase activation [159,160]. 

Therapeutics approaches have targeted IL-1β in diabetes. For example, in uninephrectomized db/db mice, an anti-IL-1β antibody reduced fibrosis, podocyte injury, and progressive decline of eGFR [132]. In the clinical field, the studies analyzed the role of IL-1β blockade in renal disease are not conclusive. In the CANTOS study (Canakinumab Anti-inflammatory Thrombosis Outcome Study), the blockade of IL-1β with canakinumab diminished inflammatory markers (hsCRP and IL-6) and the cardiovascular risk in atherosclerosis patients, but did not reduce the incidence of diabetes [161,162]. The treatment with canakinumab did not show substantive benefits on eGFR, serum creatinine, and UACR [162]. On the other hand, Cavelti-Weder et al. showed that the treatment with gevokizumab, a novel human-engineered monoclonal anti-IL-1β antibody, improved glycemia by restoring insulin production/action and reduced inflammation in patients with T2DM [163]. Another clinical trial in T2DM patients showed that the IL-1β-receptor antagonist anakinra improved glycemia and β-cell function and reduced systemic inflammatory markers [164]. The new IL-1β inhibitor rilonacept reduced systemic inflammation in patients with CKD [165], but not in T1DM patients [166]. All these results showed the key role of IL-1β in the development and progression of diabetic disease, but new studies are needed to confirm the clinical significance of this finding. 

#### 4.3.4. IL-18

Interleukin-18 (IL-18) is a member of the IL-1 cytokine family activated by inflammasome [167] that induces the expression of another cytokines and proinflammatory genes associated with apoptosis, oxidative stress, angiogenesis and cellular adhesion, such as NOX-4, p53, Il-8, TNF-α, VEGF and ICAM-1 [167,168,169]. IL-18 expression is upregulated by activation of the MAPK signaling pathway in tubular epithelial cells of diabetic subjects [170]. IL-18 increases the maturation of T lymphocytes and natural killer cells, as well as the production of other proinflammatory cytokines in obesity-induced systemic inflammation [171]. Among the different cytokines involved in DN, IL-18 seems to be the most specifically associated with these metabolic and cardiovascular risk factors [172,173]. In renal biopsies of diabetic patients, IL-18 levels were found mainly increased in proximal tubular epithelial cells [170]. IL-18 has been described as a good serum and urinary predictive marker for DN [170,173]. Moreover, urinary levels of IL-18 were positively correlated with albuminuria and kidney injury progression in T2DM individuals, suggesting a close relationship with disease progression [142,174,175]. Two studies have analyzed the efficacy of GSK1070806, a humanized IgG1/kappa antibody against IL-18 in obese and T2DM patients, but there are no studies performed in DN [176,177].

#### 4.3.5. IL17A

IL-17A is a cytokine produced by multiple cell types, including CD4+αβ T cells, γδ T cells, natural killer cells, neutrophils, macrophages, dendritic cells, lymphoid tissue inducer cells, mast cells and plasma cells [178]. IL-17 proteins, including IL-17A and IL-17F, interact with its receptors (IL-17RA–IL-17RE) and trigger downstream signaling pathways associated with inflammatory response, such as NF-κB, and oxidative stress [178,179]. IL-17A has been involved in the pathogenesis of immune and inflammatory diseases, including cardiovascular and renal diseases [178,180]. Previous studies described that Treg/Th17 ratio in T1DM patients was dysregulated, and those patients with reduced β-cell function showed increased levels of IL-17A+ in comparison with the number of Tregs, CD4+ T cells, and CD8+ T cells [181,182]. Recent studies in diabetic patients demonstrated that serum levels of IL-17A and IL-2 (Th17-associated cytokines) were increased when compared to healthy individuals [183,184]. 

In experimental models of DN, there are opposite results about the role of IL-17 in the renal diabetic pathology [185]. The gene blockade of IL-17A in mice increased the severity of renal damage induced by STZ injection. In the same study, the low dose administration of IL-17A and IL-17F in Akita diabetic mice reduced renal damage and recovered renal function via inhibition of the STAT3 signaling pathway [133]. Nevertheless, other reports showed opposite results, demonstrating that the systemic administration of IL-17A increased levels of blood pressure and inflammatory cell infiltration in the renal tissue in mice [186]. Moreover, other studies found increased IL-17A levels in serum from hypertensive patients [187]. A recent study by Lavoz et al. in BTBR ob/ob mice showed that the blockade of IL-17A with a neutralizing antibody improved albuminuria and renal pathological effects induced by diabetes [134]. Currently, some clinical trials are studying the role of IL-17A blockade with neutralizing antibodies such as secukinumab and ixekizumab in chronic human inflammatory diseases, including ankylosing spondylitis (NCT01358175; NCT01649375) [188], chronic plaque psoriasis (NCT01107457) [189] and psoriatic arthritis (NCT02404350) [190]. In the case of IL-17F, this isoform has been identified as a circulating inflammatory protein associated with increased risk of the progression of renal injury in T1DM and T2DM, among others [10]. However, there are controversial results about the serum levels of IL-17 and the severity of renal damage. Interestingly, circulating IL-17A levels were diminished in T2DM patients with or without DN when compared with normal glucose tolerance subjects [191]. Besides, plasma and urine IL-17A levels were reduced in patients with advanced DN and macroalbuminuria [133]. This data is opposite to the results obtained in a report in patients infected with cirrhotic hepatitis C virus. In this study, the circulating levels of IL-17A were increased in T2DM cohort [192]. Due to these controversial results, more studies about the role of IL17 signaling pathways in DN are needed. 

### 4.4. Other Cytokines/Proinflammatory Proteins in DN

Among several proinflammatory proteins involved in the development and progression of DN is also the cytokine IL-8, which mainly attracts neutrophils and induces oxidative stress, favoring vascular permeability and endothelial damage in the diabetic kidney [193,194]. Genetic polymorphism of IL-8 (IL8-rs4073) showed a strong association in Asian people with DN [195]. Other studies showed elevated circulating IL-8 levels in T2DM patients [193]. A bioinformatic analysis in endothelial precursor cells isolated from patients with T2DM showed that IL-8 and CXCL1 genes were the most expressed molecules in diabetic samples [196].

Tumor necrosis factor-like weak inducer of apoptosis (TWEAK) is a member of the TNF superfamily. Recently, the expression of TWEAK and its canonical receptor Fn14 was found dysregulated in the ZSF1 DN rat model, correlating with proteinuria and inflammation (CCL2 levels) [197]. Serum sTWEAK levels gradually decreased in T2DM patients in line with DN progression [193]. In another study, a negative correlation has been described between TWEAK and YKL-40, a potential biomarker for early diagnosis of incipient DN [198]. In a clinical trial in T2DM hypertensive patients with proteinuria, the combined therapy with RAAS blockers and calcium channel blockers normalized proteinuria, sTWEAK and PTX3 levels [199].

IL-20 is a pleiotropic cytokine associated with the inflammatory response that is expressed in the epithelium, endothelial cells, and monocytes/macrophages and is related to renal damage [200,201]. Hyperglycemia, ROS and TGF-β1 upregulated IL-20 mRNA/protein expression in podocytes of STZ-diabetic mice [135]. Deficiency in IL-20R1 and blockade of IL-20 with anti-IL-20 mAb 7E reduced UACR, mesangial cell expansion, and podocyte apoptosis in experimental diabetes. In the same study, experiments in cultured podocytes stimulated with IL-20 showed an increase in MMP-9, CCL2, TGF-β1, and VEGF gene expression levels [135,200]. Finally, serum IL-20 levels in DM patients were significantly elevated compared to healthy controls [135].

Gremlin, a bone morphogenetic protein 7 (BMP-7) antagonist, was identified as one of the developmental genes differentially expressed in diabetic kidneys [202]. In DN experimental models, the gremlin blockade ameliorates renal damage [203], and tubular overexpression of Gremlin in transgenic mice aggravates glomerular and tubulointerstitial injury [204] suggesting that Gremlin could be a novel therapeutic target for DN. In human biopsies of DN, Gremlin expression correlated with the grade of tubulointerstitial fibrosis and inflammation, colocalizing with TGFβ1 overexpression and Smad pathway activation [205]. Gremlin binds to vascular endothelial growth factor receptor 2 (VEGFR2) in endothelial and tubular epithelial cells, regulating the tubular epithelial to mesenchymal transition (EMT), and therefore, could contribute to renal fibrosis [206]. Gremlin activates the VEGFR2 signaling pathway in the kidney, eliciting a downstream mechanism linked to the renal inflammatory response [207]. Recently, VEGFR2 blockade, using a VEGFR2 kinase inhibitor, improved renal function, glomerular damage (mesangial matrix expansion, GBM thickening, and podocyte injury) and tubulointerstitial inflammation in T2DM model BTBR ob/ob [208]. 

Progression of DN has been also related to migration inhibitory factor (MIF), a cytokine that takes part in immune/inflammatory pathological response in DN, mainly associated with macrophage accumulation/activation [209]. In the KORA S4 study, serum MIF levels were increased in db/db mice [36] and T2DM patients [210]. Besides, increased levels of CD74 (MIF receptor) have been also reported in clinical and preclinical reports [211]. Wang et al. described that the pharmacological blockade of MIF with ISO-1 in db/db mice induced a significant decrease in albuminuria, blood glucose levels, extracellular matrix deposition, EMT, and macrophage activation in damaged kidneys [136]. Moreover, the use of the MIF antagonist (p425) in STZ-induced diabetic rats, decreased the loss of renal function (protein excretion, UACR, and serum BUN and creatinine levels) [137]. 

Macrophage inflammatory protein-1 (MIP-1), a chemokine produced by macrophages after the recognition of pathogens, is predominantly elevated in the early stages of DN [212]. Multiplex analyses of urinary inflammatory proteins revealed that levels of MIP-1α and MIP-1β, along with other chemokines and cytokines, were significantly increased in microalbuminuric DN patients compared to healthy controls [213], although only IP-10 and CCL2 correlated with albumin excretion rate and eGFR [213]. 

Other clinical trials are now targeting cytokines (IL-33) and growth factors (TGFα/β) by using humanized monoclonal antibodies in patients with DN (NCT04170543, NCT01113801, NCT01774981).

## 5. Inflammatory Intracellular Signaling Pathways

**Figure 3:**
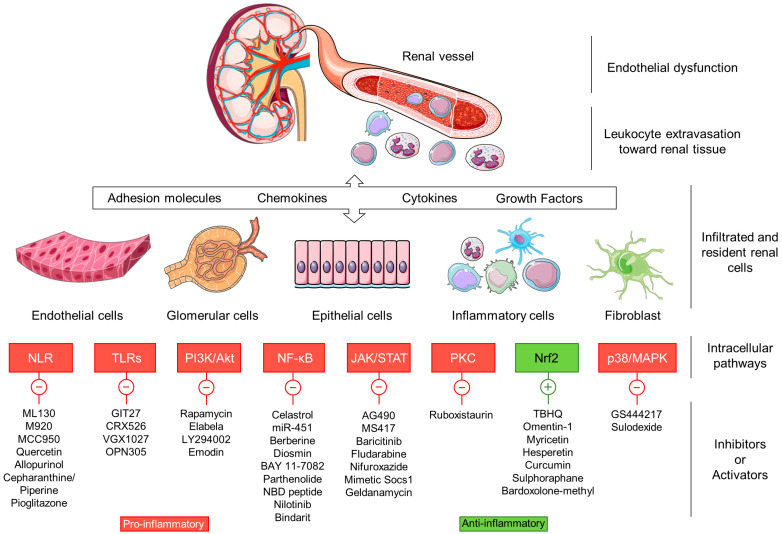
Therapeutic compounds targeting proinflammatory intracellular signaling pathways in DN.

Dysregulation of several signaling pathways leads to persistent inflammation and has been found to be involved in the onset and progression of DN (Figure 3).

### 5.1. Nod-Like Receptors

Nucleotide-binding oligomerization domain (NOD)-like receptors (NLR) are pattern-recognition cytoplasmic receptors that link innate immunity, inflammation and metabolism in diabetes [214]. NLRs are classified into four functional categories: autophagy, signal transduction, transcription activation and inflammasome formation [214,215]. Nucleotide-binding oligomerization domain-containing protein 1 (NOD1) and NOD2 are critical members of the signal transduction pathway that contribute to inflammation in DN [216]. High glucose concentrations increased NOD1, NF-κB and IL-1β expression in cultured mesangial cells, an effect reverted with the NOD1 inhibitor ML130 [217]. On the other hand, NOD2 is highly expressed in renal biopsies from diabetic patients, correlating with the severity of the disease. In the same study, hyperglycemia activated NOD2 expression in glomerular vascular endothelial cells, which promoted endothelial-to-mesenchymal transition in a MEK/ERK-mediated way [218]. Oxidative stress also activates NOD2 expression post-transcriptionally throughout the RNA-binding protein human antigen R [219]. AGEs, TNF-α, and TGF-β are also involved in NOD2 overexpression by podocytes [216]. Reduced nephrin expression was noted in kidney form NOD2-knockout mice with a high-fat diet/STZ-induced diabetes [216]. 

The NLRP family, pyrin domain-containing protein (NLRP) is composed of NLRP1, NLRP3, NLRP6 and NLRC4, which are key components of different inflammasomes [215]. Inflammasome has been implicated in metabolic disorders such as diabetes, obesity and atherosclerosis. In particular, the activation of the NLRP3 inflammasome participates in the production and persistence of inflammatory response in DN [220]. Previous in vivo and in vitro studies showed that hyperglycemia increases the expression of NLRP3 and activates caspase-1, inducing the release of IL-1β and IL-18 [221,222]. In renal tubular epithelial cells stimulated with high glucose or transforming growth factor-β1 (TGF-β1), knockdown of NLRP3 reduced ROS production and prevented EMT by inhibiting the phosphorylation of Smad3, p38/MAPK and ERK-1/2 [223]. Moreover, a study in 135 diabetic subjects with or without DN, revealed that CASP1 mRNA expression was significantly associated with decreased eGFR and severity of DN [224]. 

The gene blockade of NLRP3 in diabetic mice improved renal function, glomerular hypertrophy, glomerulosclerosis, mesangial expansion and inflammation as well as the activation of Smad3 signaling pathway [225]. Wang et al. showed that the overexpression of renal inflammasome components NLRP3, apoptosis-associated speck-like protein and caspase-1 in a STZ-rat model caused an increase in hyperuricemia, hyperlipidemia as well as elevated levels of IL-1β and IL-18. Also, treatment with the flavonoid quercetin and the uric acid-lowering drug allopurinol, reduced the expression of these inflammatory markers and ameliorated kidney damage [226]. On the other hand, the use of the anti-inflammatory compounds such as cepharanthine and piperine in a rat model of DN ameliorated hyperglycemia and renal dysfunction, and also decreased NLRP3 and NF-κB expression in diabetic kidneys [227]. In the diabetic apolipoprotein E knockout (apoE-/-) mice, administration of PPAR-γ agonist pioglitazone downregulated the expression of AGEs, RAGE, and NF-κB, and also diminished NLRP3, caspase-1, IL-18, and IL-1β levels [228]. MCC950, a selective inhibitor of NLRP3 andM920, a broad specificity caspase inhibitor, improved renal function, GBM thickening, podocyte injury, renal fibrosis, and NLRP3-dependent markers in db/db mice [229,230]. 

### 5.2. Toll-Like Receptors

Recent studies highlight the essential role of toll-like receptors (TLRs), especially TLR2 and TLR4, in DN. TLRs induce renal injury and fibrosis via the NF-κB signaling pathway [231]. Therefore, reducing the inflammatory response associated with TLRs may provide a new therapeutic approach in diabetic kidney disease. In a mouse model of advanced DN, administration of the TLR4 antagonist CRX526 significantly decreased albuminuria, blood urea nitrogen, glomerular hypertrophy, glomerulosclerosis and tubulointerstitial injury, an effect related to the impairment in CCL2/CCL5 levels, TGFβ and NF-κB activity [232]. Mixed approaches are also being tested, such as the administration of GIT27 (VGX-1027), a TLR4 and TLR2/6 signaling pathway modulator, that effectively improved insulin resistance and protected from renal injury in a db/db mice by reducing proinflammatory cytokine synthesis and oxidative stress [233]. Besides TLRs antagonists, several anti-TLR antibodies are being developed and tested. OPN305, a humanized monoclonal antibody against TLR2, is currently in phase II of a clinical trial that analyzes the prevention of delayed graft function (NCT01794663). Currently, no clinical studies are targeting TLRs signaling in diabetic kidney disease.

### 5.3. PI3K/AKT/mTOR 

Phosphatidylinositol 3-kinase/protein kinase B/mammalian target of rapamycin (PI3K/Akt/mTOR) signaling pathway regulates metabolism, proliferation, cell cycle, and protein expression, and its imbalance is involved in the development of obesity and diabetes [234]. However, the role of this pathway in DN is still controversial, suggesting the importance of discriminating the contribution of single PI3K isoforms in the diabetic kidney [235]. 

Recent studies indicate that hyperglycemia activates PI3K/Akt/mTOR, leading to glomerular hypertrophy, podocyte damage, and progressive deterioration of kidney function [236,237]. Specific PI3K/Akt/mTOR activation in podocytes generates proteinuria and mesangial cell expansion [238]. Inhibition of this pathway with rapamycin decreases macrophage infiltration and CCL2 release [239]. Furthermore, in a mouse model of diabetes, the treatment with mangiferin reduced IL-6, TNF-α, and Il-1β expression by inhibiting PTEN/PI3K/Akt pathway [240].

On the other hand, treatment with the peptide hormone Elabela activated PI3K/Akt/mTOR and reduced renal inflammation in diabetic mice by decreasing CCL2, ICAM-1, and TNF-α production [241]. In the same line, an engineered partial agonist of fibroblast growth factor-1 (FGF1^ΔHBS^) showed antioxidative effects and anti-inflammatory activities in db/db mice and also reduced oxidative stress and proinflammatory gene expression in podocytes challenged with high glucose by activating PI3K/Akt signaling [242]. Resveratrol has also shown to prevent experimental DN by regulating PI3K/Akt components in kidney tissue [243]. Likewise, in HK-2 tubular cells under high glucose conditions, treatment with the flavonoid apigenin reduced the release of TNF-α, IL-1β, and IL-6 via PI3K/Akt. The PI3K/Akt inhibitor LY294002 also reversed TNF-α reduction in these cells [244]. Moreover, in an experimental model of DN in rats, the treatment with emodin reduced IL-6 and TNF-α expression by the activation of PI3K/Akt/GSK-3β pathway [245]. Although several PI3K inhibitors have been approved for clinical use against malignant hematopoietic diseases, none of these drugs have been tested so far in the treatment of human diabetes mellitus and its complications [235].

### 5.4. Nuclear Factor-κB

NF-κB (nuclear factor-kappa B) is a ubiquitous transcription factor that modulates the expression of chemokines, cytokines, and adhesion molecules. Inhibition of NF-κB has been addressed through the use of molecules that target receptors, upstream activating kinases, protein degradation, adapter molecules, nuclear translocation, and DNA binding [246]. Numerous studies focus on the potential beneficial effects of NF-κB inhibition on the pathophysiological processes associated with DN [247]. This is the case of celastrol, a pentacyclic triterpenoid isolated from *Tripterygium wilfordii* and *Celastrus regelii* root extracts, which has shown beneficial effects on insulin resistance, body weight, renal injury, proinflammatory cytokine levels and mesangial matrix cell expansion through NF-κB inhibition [248]. In another study, intraperitoneal administration of miR-451 reduced NF-κB activity and improved microalbuminuria, glomerular damage and blood glucose levels in a DN animal model [249]. The inhibition of NF-κB by berberine (alkaloid of the isoquinoline family isolated from *Cortex phellodendri* and *Coptidis rhizome*) reduced the accumulation of extracellular matrix in the kidney, decreasing the levels of TGF-β1, ICAM-1, fibronectin and improving renal function [250]. Administration of diosmin, a flavonoid derivative, inhibited NF-κB signaling, and reduced renal levels of proinflammatory cytokines and oxidative stress in an alloxan-induced DN model [251]. On the other hand, selective blockade of IκB kinase (IKK) complex using the IKKα/β inhibitors (BAY 11-7082, parthenolide), IKKγ NBD inhibitory peptide, and BCR-ABL tyrosine kinase inhibitor (nilotinib) also had renoprotective effects in experimental models by reducing NF-κB activation, cytokine levels and oxidative stress and improving antioxidant defenses [252,253,254,255]. Recently, we have observed that inhibition of heat shock protein 90, a molecular chaperone required for stabilization/activation of IKK complex, led to a decreased expression of proinflammatory NF-κB target genes and ameliorated albuminuria, renal inflammation and fibrosis in diabetic mice [256]. In the clinical setting, bindarit an anti-inflammatory small compound that inhibits p65 and p65/p50-mediated CCL2 transcription, is being evaluated as a potential therapy for DN in association with irbesartan, but the results of this phase II clinical study have not yet been published (NCT01109212). However, it is necessary to consider the complexity of the signaling pathway associated with NF-κB and the diversity of the processes modulated by this transcription factor, which could complicate its use as a therapeutic target in DN.

### 5.5. JAK/STAT

The Janus kinases (JAK) family is comprised of four JAK tyrosine kinase receptors (JAK1, JAK2, JAK3, and TYK2), while seven members of the signal transducers and activators of transcription (STAT) family have been identified (STAT1-4, 5a, 5b and 6) [257]. These transcription factors can homo- or hetero-dimerize and activate the transcription of proinflammatory target genes [258]. Although JAK/STAT signaling actions are mainly regulated by phosphorylation of tyrosine and serine residues, “nonphosphorylated STAT” functions have also been described by several authors, as well as the epigenetic regulation of JAK without STATs mediation [259,260]. Unlike other signaling pathways, the regulation of the JAK/STAT is recognized for its simplicity, nevertheless, the wide capacity to interrelate with other cell signaling pathways such as PI3K/Akt/mTOR and MAPK/ERK axis, complicate their intracellular activity [261,262].

Among the various actions attributed to the JAK/STAT pathway, its involvement in inflammatory-based diseases appears to be inherent. Due mainly to being a major effector pathway of cytokines and others inflammatory mediators, modulation of JAK/STAT signaling has resulted in significant clinical advances in the oncology field and also in immune disorders such as rheumatoid arthritis, systemic lupus erythematosus and psoriasis [257,263]. 

JAK/STAT pathway is involved in the pathogenesis of DN [264,265]. Clinical and experimental studies demonstrate JAK1-3, STAT1, and STAT3 overactivation in the progression of DN [14,266]. Deleterious effects of JAK/STAT overactivation are mainly produced by the gene expression of cytokines, chemokines, adhesion molecules, transcription factors, growth factors, extracellular matrix proteins, pro-oxidant enzymes and scavenger receptors associated with fatty acid uptake, inflammation, oxidative stress, lipid accumulation, lipotoxicity and fibrosis [264,266,267,268]. 

Selective compounds targeting JAK2 (AG-490/tyrphostin) [269], JAK1/2 (/baricitinib) [270,271], STAT1 (fludarabine) [272] and STAT3 (nifuroxazide, S3I-201) [273] reduced albuminuria, inflammatory infiltrate, renal damage (mesangial expansion, oxidative stress, tubular atrophy, and fibrosis) and serum amyloid A in experimental DN. New design compounds such as the bromodomain inhibitor MS417 have shown the capacity to directly target acetyl-lysine residues of STAT3 and to reduce proteinuria and kidney damage in db/db mice [274]. Heat shock protein inhibitor geldanamycin also disrupts JAK/STAT signaling pathway and reduced renal damage in diabetic mice [256]. Several oral small-molecule inhibitors targeting JAK proteins (e.g., ruxolitinib, tofacitinib and baricitinib) to prevent STAT phosphorylation have been described as potential therapeutic targets for clinical use in autoimmune and inflammatory diseases [275]. Results from a phase II clinical trial demonstrate the efficacy of baricitinib (JAK1/2 inhibitor) to reduce albuminuria and improve inflammatory biomarkers in T2DM patients with high risk of progressive diabetic kidney disease [276].

The suppressors of cytokine signaling (SOCS) proteins are described as endogenous regulators of the JAK/STAT pathway and, therefore, a feasible therapeutic strategy for diabetic complications [261,266,277]. The SOCS family is comprised of eight members (SOCS1-SOCS7 and CIS), with different degrees of homology and variable N-terminal domain. The presence of the kinase inhibitory region (KIR) located at the N-terminal region of SOCS1/3 has a powerful regulatory action on JAK activity and offers an opportunity to develop novel selective kinase inhibitors mimicking KIR effect [278]. Optimization of endogenous protective resources through peptidomimetics of SOCS1 could have therapeutic advantages associated with the adverse effects observed in treatments with JAK inhibitors [276] (e.g., anemia); however, for this objective, it is necessary to carry out efficacy and safety studies in humans [279]. In the diabetes context, our findings demonstrate that SOCS1 mimetics prevent the development of retinopathy and atherosclerosis in mice [280,281]. Different SOCS1/3 delivery systems (adenovirus and cell-permeable peptide) alleviate the albuminuria and pathological renal features observed in experimental diabetic models, reducing renal inflammation, oxidative stress, and glomerular and interstitial fibrosis [261,266,282,283]. In summary, JAK/STAT activation is a key cell-signaling pathway in DN. Targeting JAK/STAT/SOCS axis could be a selective treatment in DN patients and high inflammatory risk.

### 5.6. Protein Kinase C

Hyperglycemia activates metabolic pathways through the protein kinase C (PKC), a family of enzymes widely described in DN progression [8]. In DN, hyperglycemia-mediated PKCβ and PKCδ activation in the renal cortex leads to subsequent activation of NF-κB and release of IL-6, and TNF-α by endothelial and mesangial cells [284,285]. Genetic deletion of PKCβ and PKCδ decreased renal hypertrophy, podocyte apoptosis, endothelial dysfunction, fibrosis, and proteinuria in diabetic mice [285,286]. Similarly, pharmacological inhibition of PKCβ isoform with ruboxistaurin reduced glomerular TGF-β expression and decreased fibronectin and collagen IV deposition fibrosis in experimental DN [287,288]. This approach also provided renoprotective effects in several clinical trials on patients with diabetes or diabetic complications [289] (NCT00297401; NCT00044421). 

### 5.7. Nrf2

Nrf2 (nuclear factor (erythroid-derived 2)-like 2) is a crucial protein in redox balance. Its activation triggers a powerful antioxidant response by synthesizing more than 250 genes [290]. Oxidative stress is closely linked to inflammation in diabetes mellitus and DN [291]. Nrf2 activation ameliorates pancreatic cell damage and its suppression aggravates cellular death in diabetic mice [292]. Treatment with Nrf2 activators, tert-butylhydroquinone (TBHQ) [293], bardoxolone methyl analog [294], or sulforaphane [295], reduced expression of proinflammatory cytokines and macrophage infiltration in preclinical models of DN. Furthermore, other compounds with the ability to promote Nrf2 translocation, such as omentin-1, and natural flavonoids (myricetin, hesperetin, and curcumin), improve renal function and reduces proinflammatory cytokine production in DN [296,297,298,299]. Particularly, dietary supplementation with curcumin increased the antioxidant capacity, reduced microalbuminuria and plasma MDA levels of patients with DN [300,301]. A number of clinical trials with Nrf2 inducers have been performed in patients with DN. This is the case of bardoxolone methyl, an oral antioxidant drug that activates Nrf2. A first 8-week study with 20 patients determined that this drug was able to increase the eGFR [302]. The continuation of this research, in a phase II study with 227 patients treated during 24 and 52 weeks, determined that this effect was maintained over time [303] (NCT00811889). A phase III trial with 2185 patients with T2DM and stage 4 CKD confirmed that bardoxolone methyl treatment increased eGFR; however, this study was interrupted because bardoxolone methyl treated patients showed an increased risk of cardiovascular events [304] (NCT01351675). Increased eGFR remained elevated for up to four weeks after cessation of treatment, suggesting a lower risk of end-stage renal disease [305]. Two Japanese clinical trials in diabetic patients with CKD are now testing the effectivity of bardoxolone (TSUBAKY NCT02316821, and AYAME NCT03550443). After an exploratory study in patients with rare kidney diseases (PHOENIX, NCT03366337), ongoing bardoxolone trials are being conducted in patients with Alport syndrome (CARDINAL, NCT03019185, and EAGLE, NCT03749447), or are recruiting patients with autosomal dominant polycystic kidney disease (FALCON, NCT03918447).

### 5.8. p38/MAPK

P38 mitogen-activated protein kinases are a class of mitogen-activated protein kinases (MAPK) that participates in several cellular stress responses [306]. Studies in human biopsies and cell cultures stimulated with high glucose or AGEs, demonstrated activation of the p38/MAPK pathway in podocytes, tubular, and interstitial cells [306,307]. Selonsertib, a MAP3K5 (ASK1) inhibitor and sulodexide, a sulfated glycosaminoglycan, ameliorated kidney damage by targeting p38/MAPK in T1DM and T2DM preclinical models [308,309,310]. A phase III clinical trial with selonsertib has been recently approved in DN patients (NCT04026165). 

The selected preclinical models targeting inflammatory intracellular signaling pathway and clinical trial targeting inflammation in DN are summarized in the Table 2 and Table 3, respectively.

## 6. Novel Antidiabetic Drugs with Anti-Inflammatory Actions in DN

Sodium-glucose cotransporter-2 (SGLT2) inhibitors reduce renal tubular glucose reabsorption, decreasing diabetes-associated hyperglycemia [311]. Recent clinical trials have demonstrated that new class of oral glucose-lowering therapy improved renal and cardiovascular outcomes of T2DM patients [312,313]. Recent data have also suggested that SGLT2 inhibitors (SGLT2i) possess beneficial actions against inflammatory response in DN. Preclinical models Akita or db/db mice treated with dapagliflozin reduced macrophage infiltration in the kidney and decreased inflammatory factors, such as TGF-β, CCL2, osteopontin, and ICAM-1 [314]. In db/db mice, the use of empaglifozin reduced renal inflammation determined by the reduction of NF-κB, CCL2, IL-6 levels and immune cell infiltration [315]. In the clinical field, some studies were developed about the role of SGLT2i in diabetic patients. The EMPA-REG OUTCOME (NCT01131676) trial in T2DM patients at high risk of CVD, described that the treatment with empagliflozin diminished the incidence DN and the loss of renal function [312]. The CANTATA-SU (NCT00968812) study in T2DM patients treated with metformin and canagliflozin, showed a reduction in TNFR1, IL-6, metalloproteinase-7 and fibronectin-1 levels, suggesting that canagliflozin contributes to reverse molecular processes related to inflammation, extracellular matrix turnover and fibrosis [316].

Another pillar of current treatment for diabetes is based on incretin modulators [317]. Dipeptidyl peptidase-4 inhibitors (DPP4i) are effective for the treatment of residual proteinuria in DN, a fact that is evidenced in a recent meta-analysis of the effects of sitagliptin in T2DM patients [318]. Sitagliptin also display in vitro and in vivo nephroprotective actions, including antioxidant [319,320], lipotoxicity-mediated inflammation [321] and antifibrotic actions [322]. 

GLP-1 receptor agonists (GLP-1 RA) are used in the metabolic control of obesity and T2DM patients because they enhance glucose-dependent insulin synthesis and secretion, proliferation of β-cells, inhibition of β-cells apoptosis, delay of gastric emptying and regulation of appetite by satiety-effects with body weight reduction [323,324,325]. GLP-1 RA reduces albuminuria and histological renal damage [326], and downregulates genes related to inflammation (NF-κB, TNF-α, MCP-1) [327], oxidative stress (Nox4 and subunits gp91phox, p22phox, p47phox) [328], de novo lipogenesis/lipotoxicity (SREBP-1; ABCA1) [329] and fibrosis (α-SMA, fibronectin, collagen I) [330]. These renoprotective effects observed in T2DM and CKD patients have been validated by four major clinical trials AWARD-7 [331], LEADER [332], SUSTAIN-6 [333] and ELIXA [334]. In brief, these novel antidiabetic drugs have had a tremendous impact on the treatment of cardiovascular and renal complications of diabetic patients, there exist hundreds of publications, and they have been incorporated as recommendations in all clinical guides (Table 4).

## 7. Perspectives and Conclusions

DN is one of the most leading causes of CKD/ESRD. Established treatment for patients with diabetes includes control of blood glucose, cholesterol and hypertension, but provide incomplete protection against ESRD and cardiovascular risk. There is, therefore, an urgent need to apply more effective therapies for patients with DN.

Targeting inflammation may be useful in the prevention and treatment of DN. In this sense, the inhibition of IL-1β activity (gevokizumab, anakinra and canakinumab) and the blockade of CCL2 (AF2838) and their receptors CCR2/CCR5 (CCX140-B, PF-04634817, BMS-813160) open a new horizon for the employment of anti-inflammatory therapies in patients DN, including those with cardiovascular risk. The blockade of some adhesion molecules (Galectin-3, αVβ3) has also provided beneficial effects in DN. NLRP3 inflammasome and its related cytokine IL-18 have emerged as important inflammatory molecules involved in the progression of DN. However, no compounds targeting inflammasome components have been yet tested in clinical trials. Although Th17 response has gained attention in the last years, there are controversial results about its role in DN. On the other hand, oxidative stress is closely related to inflammation in DN. Several clinical reports suggest that activation of the Nrf2 pathway, mainly with bardoxolone, may be of interest.

As discussed in this review, several urinary and plasma inflammatory biomarkers have been examined at early and advanced stages DN. These biomarkers can be associated to glomerular and tubular damage, oxidative stress, and fibrosis development [335]. Moreover, some inflammatory markers (TNFα, IL-6 and CCL2) and adipokines (resistin, visfatin and leptin) detected in the saliva of T2DM patients could have a prognostic value on the progression of DN, given their high correlation with serum/urinary inflammatory markers [336,337]. The refinement of -omic techniques and novel bioinformatic platforms predict an exponential growth in this area, both in the diagnosis and categorization of DN.

In conclusion, emerging therapies for DN are focused on the modulation of inflammatory pathways that control the functional and structural abnormalities in DN. Compounds targeting some chemokines and their receptors, cytokines, NF-κB, NLRP3 inflammasome, PI3K/Akt and JAK/STAT pathways, and the Nrf2-regulated antioxidant defense have shown remarkable efficacy in preclinical studies. In this regard, some incipient clinical trials (e.g., baricitinib, canakinumab) have afforded promising results in patients with DN. Therefore, although recent clinical trials with SGLT2i and GLP-1 RA have provided beneficial effects in patients with DN, additional specific anti-inflammatory strategies may be of special interest to further reduce residual proteinuria and progression to renal failure. Further research is needed to find the balance between the benefits and potential risks of these incoming therapies, and to identify the subgroups of patients most likely to benefit from them.

## Figures and Tables

**Table 2:** Selected preclinical studies targeting inflammatory intracellular signaling pathways in DN.

Target	Diabetic Model	Strategy	Category	Conclusion	Ref.
**NOD- like receptor**	SD rats + STZ (55 mg/kg)	Quercetin or allopurinol	Flavonoid or XO inhibitor	Ameliorates hyperuricemia and reduces IL-1β and IL-18 levels.	[226]
	SD rats + STZ (50 mg/kg)	Cepharanthine and Piperine	Nutraceuticals	Decreases cytokines NLRP3-dependent.	[227]
	ApoE-/- mice + STZ (80 mg/kg)	Pioglitazone	PPARγ agonist	Restores renal function. Downregulates AGEs, RAGEs and cytokines NLRP3-dependent.	[228]
	db/db mice	MCC950	Selective NLRP3 inhibitor	Ameliorates GBM thickness, podocyte injury and renal fibrosis.	[229]
	db/db mice	M920	Pan-caspase inhibitor	Reduces NLRP3-inflammasome (IL-1β, IL-18, NLRP3, caspase-1) and profibrotic markers.	[230]
**TLRs**	eNOS^−/−^ + STZ mice	CRX-526	TLR4 antagonist	Ameliorates histological changes, inflammatory and profibrotic markers.	[232]
	db/db mice	GIT27	TLR4 inhibitor	Reduces proinflammatory, oxidative stress and lipid metabolism markers.	[233]
**PI3K/** **AKT**	SD + STZ (60 mg/kg)	Rapamycin	mTOR inhibitor	Ameliorates histological changes, inflammation and fibrotic markers.	[239]
	C57BL/6 + STZ (60 mg/kg)	Mangiferin	Natural phenolic xanthonoid	Reduces inflammation, oxidative stress and profibrotic markers.	[240]
	C57BL/6J + STZ (50 mg/kg)	Elabela	Peptide agonist Apelin/APJ receptor	Reduces GBM thickening, podocyte damage, inflammation, apoptosis and fibrosis markers.	[241]
	Wistar rats + STZ (60 mg/kg)	Emodin	Derived from root rhubarb	Attenuates inflammation, oxidative stress and apoptosis markers.	[245]
**NF-κB**	db/db mice	Celastrol	Celastrus regelii root extracts	Improves lipid accumulation, oxidative stress and proinflammatory markers.	[248]
	db/db mice	miR-451	LMP7 modulator	Decreases histological lesions, inflammation and fibrosis markers.	[249]
	Wistar rats + STZ (65 mg/kg)	Berberine	Flavonoid	Improves systemic and renal cortex inflammatory response.	[250]
	SD rats + STZ (50 mg/kg).	BAY 11-7082	Phospho-IκB inhibitor	Reduces proinflammatory cytokines and oxidative stress markers.	[252]
	ApoE^−/−^ mice + STZ (125 mg/kg -2 days)	NBD peptide	Mimetic IKKβ-NEMO complex	Decreases histological lesions, inflammation and fibrosis.	[254]
	SD rats + STZ (50 mg/kg)	Nilotinib hydrochloride	BCR-ABL tyrosine kinase inhibitor	Reduces oxidative stress, inflammation and profibrotic marker expression.	[255]
**JAK/** **STAT**	SD rats + STZ (60 mg/kg)	AG-490 (Tyrphostin)	JAK 2 inhibitor	Prevents JAK2 and STATs phosphorylation.	[269]
	Akita mice + JAK2 Pod-overexpression	Tyrphostin or Baricitinib	JAK 2 inhibitors	Ameliorates histological changes, serum amyloid A3 plasma and kidney tissue.	[228,229]
	SD rats + STZ (50 mg/kg)	Nifuroxazide	STAT3 inhibitor	Diminishes inflammatory and profibrotic factors and infiltrating cells.	[273]
	Double db/db- SIRT-1^−/−^ mice	MS417	BET-specific BRD4 inhibitor	Attenuates histological changes and STAT3 activity.	[274]
	CD-1 mice + STZ (150 mg/kg)	Gene delivery SOCS1	SOCS1 Overexpression	Attenuates renal hypertrophy, inflammation and profibrotic markers.	[282]
	ApoE^−/−^ + STZ (125 mg/kg)	SOCS1 delivery system	Mimetic SOCS1- KIR region	Reduces atherosclerosis, structural changes and inflammatory markers.	[219,241]
**PKC**	Wistar rats + STZ (55 mg/kg)	Ruboxistaurin	Selective PKCβ inhibitor	Improves histological changes and attenuates TGFβ/Smad2/3 pathway.	[287]
	Swiprosin-1^−/−^ C57BL/6 mice + STZ (150 mg/kg)	Ruboxistaurin	Selective PKCβ inhibitor	Ameliorates histological changes, apoptosis and Swiprosin-1 expression.	[288]
**Nrf2**	SD rats + IR injury + STZ (65 mg/kg)	TBHQ	Synthetic antioxidant	Ameliorates oxidative stress, inflammation and apoptosis.	[293]
	db/db and C57BL/6 mice + HFD	γ-glutamyl transpeptidase	Bardoxolone methyl analog	Reduces body weight, histological changes and GBM thickening.	[294]
	Wistar rats + STZ (58 mg/kg)	Sulforaphane	Cruciferous vegetables extracts	Diminishes inflammation, oxidative stress markers, DNA damage and cell death.	[295]
	SD rats + STZ (60 mg/kg)	Hesperetin	Flavonoid	Ameliorates histological changes, inhibition AGEs/RAGE axis and inflammation.	[296]
	C57BL/6 + STZ (50 mg/kg) and Nrf2 knockdown mice	Myricetin	Flavonoid	Mitigates inflammation, oxidative stress and fibrosis markers.	[297]
	db/db mice	Omentin-1	Adipokine	Reduces proinflammatory cytokines and oxidative stress markers.	[298]
	SD rats + STZ (55 mg/kg)	Curcumin	Flavonoid	Lipid accumulation, angiogenesis, profibrotic and podocyte damage markers.	[299]
**p38/** **MAPK**	eNOS^−/−^ mice + STZ (55 mg/kg)	GS-444217	ASK1 inhibitor (Selonsertib)	Ameliorates histological changes, inflammatory and profibrotic markers.	[308]
	eNOS^−/−^ db/db mice	GS-444217 + ACEi			[309]
	OLETF rats	Sulodexide	Sulfated GAGs	Reduces histological changes, urinary VEGF and profibrotic markers.	[310]

**Abbreviations**: STZ: streptozotocin; SD: Sprague Dawley; OLEFT: Otsuka Long-Evans Fatty Tokushima, NBD: NEMO-binding domain; SOCS: suppressor of cytokine signaling; TBHQ: tert-butylhydroquinone; ACEi: angiotensin-converting enzyme inhibitor; PPARγ: peroxisome proliferator-activated receptor gamma; XO: xanthine oxidase; LMP7: low-molecular mass protein-7; NEMO: NF-κB essential modulator; BET: bromodomains and extraterminal motif; BRD4: bromodomain containing 4: ASK1: apoptosis signal-regulating kinase 1; GAGs: glycosaminoglycans: GBM: glomerular basal membrane.

**Table 3:** Selected clinical trials targeting inflammation in DN.

Target	Compound	Enrollment	Phase	Status	1stor 2nd Outcome	Identification
**Chymase inhibitor**	BAY1142524 Fulacimstat	152	2	C	UACR	NCT03412006
**VAP-1 inhibitor**	ASP8232	55	2	C	AE—UACR	NCT02218099
**Galectin-3 antagonist**	GCS-100	375	2	U	eGFR	NCT02312050
**Anti-Human** **αVβ3**	VPI-2690B Monoclonal antibody	165	2	C	Albuminuria—eGFR	NCT02251067
**CCL2 inhibitor**	AF2838 Bindarit	100	2	C	UACR	NCT01109212
**CCL2 inhibitor**	Spiegelmer^®^ NOX-E36	76	2	C	UACR	NCT01547897
**CCR2 inhibitor**	Propagermanium	45	2	A	UACR—eGFR	NCT03627715
**CCR2 inhibitor**	CCX140-B	332	2	C	UACR	NCT01447147
**CCR2/5 antagonist**	PF-04634817	226	2	C	UACR	NCT01712061
**CCR2/5 antagonist**	BMS-813160	319	2	T	UACR	NCT01752985
**PDE inhibitor**	Pentoxifylline	196	4	R	eGFR	NCT03664414
**PDE inhibitor**	Pentoxifylline	2510	4	A	Death—ESRD—UACR	NCT03625648
**Anti-Human IL-1β**	Canakinumab	10066	3	C	MACE—UACR—eGFR	NCT01327846
**Anti-Human** **IL-18**	GSK1070806 Monoclonal antibody	37	2	C	UACR, HbA1c and metabolic changes	NCT01648153
**Anti-Human** **IL-33**	MEDI3506 Monoclonal Antibody	168	2	R	UACR	NCT04170543
**Anti-Human** **TGFβ1**	Galunisertib Monoclonal Antibody	417	2	T	UACR	NCT01113801
**Anti-Human TGFα/Epiregulin**	LY3016859 Monoclonal antibody	60	2	C	UACR	NCT01774981
**JAK2 inhibitor**	Baricitinib	130	2	C	UACR—eGFR	NCT01703234
**PKCβ Inhibitor**	Ruboxistaurin	20	3	C	UACR	NCT00297401
**NRF2 inducer**	Bardoxolone Methyl	1323	3	A	eGFR or ESRD	NCT03550443
**NRF2 inducer**	Bardoxolone Methyl	2185	3	C	ESRD or death	NCT01351675
**NRF2 inducer**	Bardoxolone Methyl	216	2	C	AE—eGFR	NCT02316821
**Anti-inflammatory inhibitor**	Colchicine	160	NA	A	UACR	NCT02035891
**Sulfated GAGs**	KRX-101 Sulodexide	1248	4	T	ESRD—UACR	NCT00130312
**ASK-1 inhibitor**	GS-4997 Selonsertib	3300	3	A	eGFR—ESRD	NCT04026165

**Abbreviations**: A: active; AE: adverse effects; C: completed; NA: not applicable; NR: not yet recruiting; R: recruiting; T: terminated; U: unknown. UACR: urinary albumin creatinine ratio; eGFR: estimated glomerular filtration rate; ESRD: end-stage renal disease; MACE: major adverse cardiovascular events; ERPF: effective renal plasma flow.

**Table 4:** Novel antidiabetic drugs with anti-inflammatory actions in patients with DN.

Target	Compound	Enrollment	Phase	Status	1st or 2nd Outcome	Identification
**SGLT2i**	Empaglifozin	70	4	R	Albuminuria—eGFR	NCT04127084
**SGLT2i**	Empaglifozin	7064	3	C	MACE and UACR	NCT01131676
**SGLT2i + DPP-4i**	Empaglifozin + Linagliptin	66	4	R	eGFR—UACR	NCT03433248
**SGLT2i + GLP-1 RA**	Empaglifozin + Semaglutide	80	4	NR	Albuminuria—eGFR	NCT04061200
**SGLT2i**	Canagliflozin	4401	3	C	eGFR—ESRD	NCT02065791
**SGLT2i**	Canagliflozin	300	3	A	eGFR—UACR	NCT03436693
**SGLT2i**	Canaglifozin	1452	3	C	HbA1c change	NCT00968812
**SGLT2i**	Dapagliflozine	44	4	C	eGFR—ERPF—UACR	NCT02682563
**DPP-4i**	Linagliptin	48	4	C	eGFR—ERPF	NCT02106104
**DPP-4i**	Linagliptin	6991	4	C	MACE—eGFR	NCT01897532
**DPP-4i**	Alogliptin	5380	3	C	MACE	NCT00968708
**DPP-4i**	Saxagliptin	18206	4	C	MACE	NCT01107886
**DPP-4i**	Sitagliptin	14671	3	C	MACE—eGFR	NCT00790205
**GLP-1 RA**	Lixisenatide	40	4	C	eGFR—ERPF	NCT02276196
**GLP-1 RA**	Exenatide	92	4	C	Albuminuria	NCT02690883
**GLP-1 RA**	Dulaglutide	577	3	C	HbA1c—eGFR—UACR	NCT01621178
**GLP-1 RA**	Liraglutide	9341	3	C	MACE and UACR	NCT01179048
**GLP-1 RA**	Semaglutide	3297	3	C	MACE—HbA1c—UACR	NCT01720446
**GLP-1 RA**	Lixisenatide	6068	3	C	MACE—UACR	NCT01147250

**Abbreviations**: A: active; C: completed; NR: not yet recruiting; R: recruiting; UACR: urinary albumin creatinine ratio; eGFR: estimated glomerular filtration rate; ESRD: end-stage renal disease; MACE: major adverse cardiovascular events; ERPF: effective renal plasma flow

## Abbreviations

ABCA1	ATP binding cassette transporter A1
ACEi	Angiotensin-converting-enzyme inhibitors
AGEs	Advanced glycation end-products
Akt	Protein kinase B
ApoE	Apolipoprotein E
AP-1	Activator protein-1
ARBs	Angiotensin Receptor Blockers
ASK1	Apoptosis signal-regulating kinase 1
BMP-7	Bone morphogenetic protein 7
BTBR	Black and tan brachyuric
BUN	Blood urea nitrogen
CASP1	Caspase-1
CCL11	Chemokine-CC motif ligand 11
CCL2	Chemokine-CC motif ligand 2
CCL5	Chemokine-CC motif ligand 5
CCR2	C-C chemokine receptor 2
CCR5	C-C chemokine receptor 5
CD206/MRC1	Mannose receptor
CD4+	Cluster of differentiation 4+
CD74	Cluster of differentiation 74
CD8+	Cluster of differentiation 8+
CKD	Chronic kidney disease
Col IV	Collagen IV
CVD	Cardiovascular Disease
CX3CL1	Fractalkine
CX3CR1	Fractalkine receptor
CXCL1	Chemokine (C-X-C motif) ligand 1
CXCL10	Chemokine (C-X-C motif) ligand 10
CXCL12	Chemokine (C-X-C motif) ligand 12
CXCL16	Chemokine (C-X-C motif) ligand 16
CXCL5	Chemokine (C-X-C motif) ligand 5
CXCL8	Chemokine (C-X-C motif) ligand 8
CXCL9	Chemokine (C-X-C motif) ligand 9
CXCR4	C-X-C chemokine receptor type 4
db/db	Diabetic/diabetic
DCs	Dendritic cells
DN	Diabetic nephropathy
DNA	Deoxyribonucleic acid
DPP4i	Dipeptidyl peptidase-4 inhibitors
eGFR	Estimated glomerular filtration rate
EMT	Epithelial to mesenchymal transition
EndMT	Endothelial to mesenchymal transition
ERK 1/2	Extracellular Signal-Regulated Kinase 1/2
ESRD	End-stage renal disease
FGF1	Acidic fibroblast growth factor
GBM	Glomerular basement membrane
GLP-1	Glucagon-Like Peptide 1
GLP-1 RA	GLP-1 receptor agonists
GSK-3β	Glycogen synthase kinase 3β
HbA1c	Hemoglobin A1c
HK-2	Human kidney 2 cells
HO-1	Heme Oxygenase 1
ICAM-1	Intercellular adhesion molecule-1
IFN-γ	Interferon-γ
IgG	Immunoglobulin G
IgM	ImmunoglobulinM
IKKα/β/γ	IκB kinase α/β/γ
IL-10	Interleukin-10
IL-17A/F	Interleukin-17A/F
IL-18	Interleukin-18
IL-1β	Interleukin-1β
IL-2	Interleukin-2
IL-20	Interleukin-20
IL-6	Interleukin-6
IL-8	Interleukin-8
IP-10	Interferon-inducible protein 10
JAK	Janus Kinase/ Just Another Kinase
KIR	Kinase inhibitory region
KRIS	Risk inflammatory signature
LDL	Low Density Lipoprotein
Ly6C	Lymphocyte antigen 6C
mAb	Monoclonal Antibody
MAP3K5	Mitogen-activated protein kinase 5
MAPK	Mitogen-Activated Protein Kinase
MDA	Malondialdehyde
MEK	Mitogen-activated protein kinase kinase
MIF	Macrophage migration inhibitory factor
MIP-1	Macrophage Inflammatory Protein-1
MMP	Matrix metalloproteinase
mTOR	Mammalian target of rapamycin
NADPH	Nicotinamide adenine dinucleotide phosphate hydrogen
NBD	Nucleotide-binding domain
NF-κB	Nuclear factor kappa-light-chain-enhancer of activated B cells
NLR	Nucleotide-binding oligomerization domain (NOD)-like receptors
NLRP	Pyrin domain-containing protein
NOD1	Nucleotide-binding oligomerization domain-containing protein 1
NOD2	Nucleotide-binding oligomerization domain-containing protein 2
NOX-4	NADPH oxidase 4
Nrf2	Nuclear factor (erythroid-derived 2)-like 2
Ob	obese
P38/MAPK	p38 mitogen-activated protein kinase
p53	Cellular tumor antigen p53
PDGF	Platelet derived growth factor
PI3K	Phosphatidylinositol 3-kinase
PKC	Protein kinase C
PPAR-γ	Peroxisome proliferator-activated receptor-γ
PTEN	Phosphatase and tensin homolog
PTX3	Pentraxin 3
RAAS	Renin–-angiotensin–-aldosterone system
ROS	Reactive Oxygen Species
SGLT2	Sodium-glucose cotransporter-2
SGLT2i	SGLT2 inhibitors
sICAM-1	Soluble intercellular adhesion molecule-1
Smad	Mothers against decapentaplegic homolog
SOCS	Suppressors of cytokine signaling
SREBP-1	Sterol regulatory element binding transcription factor 1
STAT	Signal Transducer and Activator of Transcription
sTWEAK	Soluble TNF-related weak inducer of apoptosis
STZ	Streptozotocin
T1DM	Type 1 and type 2 diabetes mellitus
T2DM	Type 2 diabetes mellitus
TBHQ	Tert-butylhydroquinone
TGF-β	Transforming growth factor-β
Th1	T helper cell type 1
Th17	T helper cell type 17
Th2	T helper cell type 2
TLRs	Toll-like receptors
TNF-α	Tumor necrosis factor-α
TNFR1	Tumor necrosis factor receptor 1
TNFR2	Tumor necrosis factor receptor 2
Tregs	Regulatory T cells
TYK2	Tyrosine kinase 2
UACR	Urine albumin to creatinine ratio
VAP-1	Vascular adhesion protein-1
VCAM-1	Vascular cell adhesion molecule-1
VEGF	Vascular endothelial growth factor
VEGFR2	Vascular endothelial growth factor receptor 2
ZSF1	Zucker Diabetic Fatty 1
α-SMA	α-smooth muscle actin
